# Learning by Population Genetics and Matrix Riccati Equation

**DOI:** 10.3390/e25020348

**Published:** 2023-02-14

**Authors:** Sergei Kozyrev

**Affiliations:** Steklov Mathematical Institute of Russian Academy of Sciences, Gubkina St. 8, 119991 Moscow, Russia; kozyrev@mi-ras.ru

**Keywords:** learning theory, statistical mechanics, evolution theory, 02.30.Hq, 68T05, 92D15

## Abstract

A model of learning as a generalization of the Eigen’s quasispecies model in population genetics is introduced. Eigen’s model is considered as a matrix Riccati equation. The error catastrophe in the Eigen’s model (when the purifying selection becomes ineffective) is discussed as the divergence of the Perron–Frobenius eigenvalue of the Riccati model in the limit of large matrices. A known estimate for the Perron–Frobenius eigenvalue provides an explanation for observed patterns of genomic evolution. We propose to consider the error catastrophe in Eigen’s model as an analog of overfitting in learning theory; this gives a criterion for the presence of overfitting in learning.

## 1. Introduction

In the present paper we discuss the relation of three different areas, such as statistical physics, learning theory, and theory of biological evolution. This relationship has been already widely discussed in the literature. In particular, the relationship between the theory of evolution and learning theory was mentioned by A.Turing [1] (learning is the minimization of risk and biological evolution is optimization of fitness). The relationship between statistical physics and evolutionary theory was discussed in [2,3]. The consideration of biological evolution as a learning problem for functional programming was discussed in [4,5,6,7,8] and various aspects of relation of statistical physics, learning and evolution was considered in [9,10,11]. In this paper, we consider the application of population genetics in learning theory.

Universal patterns of genome evolution found in genomics [2,3] were discussed by E. Koonin as a manifestation of the Gibbs distribution of a model of “interacting gas of genes”. Here, we discuss a model of population genetics given by a matrix Riccati equation, which is a generalization of the Eigen’s quasispecies model [12]. Patterns of genome evolution [2] in our model correspond to known estimates of the Perron–Frobenius eigenvalue. Eigen’s “error catastrophe” for this model takes the form of divergence of the Perron–Frobenius eigenvalue of the matrix Ricatti equation in the limit of large matrices. Error catastrophe describes the regime of ineffective purifying selection in population genetics in the case of high mutation rates. From the point of view of learning theory, the “error catastrophe” describes the transition to overfitting in the corresponding learning model.

Biological evolution was compared with the statistical physics of disordered systems (or spin glass theory, in particular, frustration in biology and spin glasses were mentioned) in [9]; these authors also discuss the relation of evolution and learning [10,11]. Let us note that in [10,11], solvability of learning problems in evolution was taken for granted and here we address exactly this problem of solvability (in the form of problem of overfitting in learning).

The exposition of this text is as follows. In Section 2 of this text we discuss the Eigen’s quasispecies model and error catastrophe; introduce our generalization of this model (a kind of matrix Riccati model); describe the error catastrophe as divergence of the Perron–Frobenius eigenvalue of the matrix Riccati model in the limit of large matrices; and describe from this point of view known patterns of genomic evolution. In Section 3, we introduce a population genetics-type learning model and discuss the relation of the error catastrophe to overfitting in learning. In Appendix A (the Appendices), the Perron–Frobenius theorem, matrix Riccati equations, and basic definitions of statistical learning theory are discussed.

## 2. Generalization of Eigen’s Model in Population Genetics

**The Eigen’s model in population genetics**. Here we consider the Eigen’s quasispecies model following [12] (for a discussion of relation to other models of population genetics see [13], in particular, Moran’s model was introduced in [14]). We investigate a family of different “genotypes” with populations $x_{i}\geq 0$, i=1,⋯,n; the total population is normed: $\sum_{i=1}^{n}x_{i}=1$.

The following system of equations [12] describes the dynamics, where *Q* is a matrix with positive matrix elements. The non-linear term describes the competition of genotypes

(1) $$\frac{d}{dt}x_{i}\left(t\right)=\sum_{j=1}^{n}Q_{ij}x_{j}\left(t\right)-E\left(t\right)x_{i}\left(t\right),\;E\left(t\right)=\sum_{i,j=1}^{n}Q_{ij}x_{j}\left(t\right).$$

Diagonal matrix elements of *Q* describe reproduction rates of genotypes, off-diagonal matrix elements describe mutation rates.

The analog of the Eigen’s model with discrete time is as follows

$$x_{i}\left(t+1\right)=\frac{1}{E(t)}\sum_{j=1}^{n}Q_{ij}x_{j}\left(t\right),\;E\left(t\right)=\sum_{i,j=1}^{n}Q_{ij}x_{j}\left(t\right).$$

In Eigen’s paper [12], genotypes are enumerated by strings of characters. The length of the genome is denoted by ν, the size of the alphabet is denoted by *k* (in particular for nucleotides k=4). The fidelity of reproduction of a single nucleotide is *q*, 0<q<1. In this case, the accuracy of reproduction of a genome is $R_{ii}=q^{\nu }$. Mutation rates in Eigen’s model are equal to

(2) $$Q_{ij}=\epsilon^{d(i,j)}R_{ii},\;\epsilon =\frac{q^{-1}-1}{k-1}.$$

Here, d(i,j) is the number of different nucleotides in the *i*-th and *j*-th genotypes (called the Hamming distance). The reproduction and mortality rates of the *i*-th genotype are denoted $P_{i}$ and $D_{i}$ correspondingly, which gives for diagonal matrix elements $Q_{ii}=P_{i}R_{ii}-D_{i}>0$.

The Eigen’s model is a matrix Riccati Equation (A2) (see Appendix A.2), where $A_{4}=Q$, $A_{1}=0$, $A_{3}=0$, $A_{2}=e^{†}Q$, and e is a vector with unit coordinates. By the Perron–Frobenius theorem, see Appendix A.1, the dynamics of this model reduces to convergence to a stationary solution (which for Eigen’s model is called quasispecies) given by the Perron–Frobenius eigenvector corresponding to the Perron–Frobenius eigenvalue of matrix *Q* (the largest eigenvalue of a matrix with positive matrix elements).

**Error threshold**. Eigen considered the behavior of the stationary solution of (1) (the quasispecies) depending on the mutation rate in the frameworks of perturbation theory by small mutation rates (i.e., by small off-diagonal matrix elements of *Q*). Let us denote *I* the most fit genotype for the PF eigenvector of *Q* (i.e., the sequence with the maximal population). Then, if the matrix *Q* is diagonal (there are no mutations), one has $x_{I}=1$ and $x_{i}=0$, i≠I. If mutations are small but non-zero (first-order perturbation of the stationary solution), one has $x_{i}=x_{i}^{\left(0\right)}+x_{i}^{\left(1\right)}$, $x_{I}^{\left(0\right)}=1$, for $i\neq I:x_{i}^{\left(0\right)}=0$, first-order corrections are given by

(3) $$\left(Q_{ii}-Q_{II}\right)x_{i}^{\left(1\right)}+Q_{iI}=0,\;x_{i}^{\left(1\right)}=\frac{Q_{iI}}{Q_{II}-Q_{ii}}.$$

The correction $x_{I}^{\left(1\right)}$ satisfies

$$x_{I}^{\left(1\right)}+\sum_{i\neq I}x_{i}^{\left(1\right)}=0,$$

therefore

(4) $$x_{I}^{\left(1\right)}=-\sum_{i\neq I}\frac{Q_{iI}}{Q_{II}-Q_{ii}}.$$

Expressions (3) and (4) give coordinates of the PF vector for the stationary solution of (1) in the first order of perturbation theory by small mutation rates.

The correction $x_{I}^{\left(1\right)}$ is small if the series for the rates of mutations is small

(5) $$\sum_{i\neq I}Q_{iI},$$

and if there are no small denominators in the above Formula (4), i.e., $Q_{II}-Q_{ii}>\delta >0$ for some δ, this condition is sufficient.

For Eigen’s choice of mutation rates (2), this series can be estimated by a geometric progression. Therefore, if the reproduction accuracy *q* is not close to one, this series will be large (actually we discuss finite but long progressions). The regime when the stationary state (the quasispecies) for Eigen’s model loses localization is called the error catastrophe. The corresponding error catastrophe mutation rate separates regimes of effective and ineffective purifying selection in population genetics.

It is easy to see that $\sum_{i}Q_{iI}$ is the estimate (A1) for the Perron–Frobenius eigenvalue for matrix *Q* (and $x_{i}=Q_{iI}$, i≠I are estimates of coordinates of the PF vector in the first order of perturbation theory, if we ignore denominators in (3)). Therefore, *Eigen’s model is a variant of the matrix Riccati equation and error catastrophe is the divergence of the Perron–Frobenius eigenvalue of the model in the limit of large matrices*.

**Generalization of Eigen’s model**. Let us introduce a generalization of Eigen’s model. We consider a space of possible genotypes and a set of possible mutations $E=[e_{1},\ldots ,e_{n}]$. Here, $e_{s}$ are not necessarily point mutations, mutations may include duplications, insertions, deletions, etc. Let us put in correspondence to a mutation $e_{s}$ a weight $w(e_{s})>0$ as “evolutionary effort” to produce the mutation. The Boltzmann factor $e^{-\alpha w(e_{s})}$ (α>0 is a parameter of the kind of inverse temperature for mutations) is the analog of the mutation rate for a single mutation 1−q in Eigen’s model. We define the transition rate from genotype *i* to genotype *j* in the model of population genetics under consideration as

(6) $$Q_{ji}=\sum_{p:i\to j}e^{-\alpha \sum_{k\in p}w\left(e_{s}\left(k\right)\right)},$$

where summation over *p* runs over paths p:i→j of generation of *j* from *i* and summation over *k* runs over mutations along the path *p* (i.e., *k*-th mutation at the path *p* is $e_{s}$). This sum over *k* weights of mutations is the analog of the Hamming distance in (2), the summation over paths takes into consideration retinal evolution (possibility to access *j* from *i* taking mutations in different order).

We define diagonal matrix elements $Q_{ii}$ by the functional *R*, which describes fitness (β>0 is the inverse temperature for selection, temperatures for selection and mutations can be different)

(7) $$Q_{ii}=e^{-\beta R[i]}.$$

Then, we define the model of population genetics by using equations of the Eigen’s model (1) with more general mutation and survival matrix (6), (7) and more general family of mutations, this allows us to explain patterns of genomic evolution (9) and (10); see the discussion below.

The condition for effective purifying selection for this model is the condition of convergence of the estimate (A1) of the Perron–Frobenius eigenvalue of the model, if we exclude in this estimate the diagonal matrix element and reduce the corresponding series to (5), we get

(8) $$Z=\sum_{j}\sum_{p:i\to j}e^{-\alpha \sum_{k\in p}w\left(e_{s}\left(k\right)\right)}.$$

This expression has the form of a statistical sum over iterated mutations. Here, *i* is the starting point of evolution (the ancestral genome). Critical phenomena for this statistical sum (transition between convergence and divergence of (8) depending on the inverse temperature α) describe the transition between regimes of effective and ineffective purifying selection in population genetics (the error catastrophe). Let us note that all possible mutations give contributions to this expression. Even if only point mutations are taken into account, this gives a contribution of order of the length of a genome. Therefore, to keep (8) small, the mutations rates should be sufficiently low. This observation also puts limitations on learning without overfitting, see the discussion in Section 3.

**Laws of genomic evolution, population genetics, and statistical physics**. Let us consider two examples of genomic evolution discussed in [2,3] and show that patterns of genomic evolution can be considered as a manifestation of the statistical sum (8).

Orthologous proteins in different species are related by common origin. For such proteins the logarithm of amino acid substitution frequency is distributed according to normal law. Let us consider for orthologous proteins the evolution by random independent amino acid substitutions with probability of substitution A→B depending only on amino acids *A*, *B*. The coordinates of the PF vector, by perturbation theory (3), can be estimated by mutation rates (6), which gives for the coordinates

(9) $$e^{-\alpha \sum_{k}E_{k}},$$

where $E_{k}$ are weights of mutations in the process of protein generation from the ancestor (summation with respect to *k* is the summation along the path of evolution). For independent mutations (this is the assumption that the evolution is neutral) we obtain the lognormal distribution for protein occurrences in the orthologous family (coordinates of the PF vector).

Genes in the same genome generated by duplication events are called paralogous. Let us consider evolution by gene duplication, each duplication corresponds to a contribution in (6) (evolutionary effort) *E*. Then, for a family of *N* paralogous genes, the “evolutionary effort” contribution is NE; thus, the expression for a coordinate of the PF vector corresponding to a family of *N* paralogous genes will be equal to

(10) $$e^{-\alpha NE},$$

i.e., one obtains the degree distribution for sizes *N* of families of paralogs.

Therefore, the statistical sum (8) explains known patterns of genomic evolution. For discussion of these patterns, E.V. Koonin conjectured [2,3] an idea of “interacting gas of genes”, i.e., the evolution of genomes should be explained by the Gibbs distribution of some model of statistical physics with interaction of genes. This hypothetical model was also called “the third evolutionary synthesis”. In [9,10,11], following these ideas, the relation between statistical physics, learning, and evolution was discussed. In these papers, the authors applied the approach of [15,16], where evolution phenomena were explained using the structure of the fitness landscape (i.e., the diagonal part of the selection–mutation matrix in the above model).

In our approach, the mutation off-diagonal part of this matrix was applied, universal genomic evolution patterns follow from the universality of the mutation matrix (6). From the point of view of learning theory, see the next Section, the universal form of mutation rates looks like universal regularization in learning. The above generalization of Eigen’s model (6) and (7) can be considered as a possible candidate for the “interacting gas of genes” model (the corresponding Gibbs distribution is (8), or the PF eigenvalue estimate).

## 3. Learning Theory and Population Genetics

Learning is the minimization of the risk functional (or loss functional) *R* over the hypothesis space F of the system, see, in particular, [17] and Appendix A.3. Analogy between learning and Darwinian evolution, i.e., between minimization of risk and optimization of fitness, was mentioned by A. Turing [1] in 1950, now this idea attracts attention [4,5,8,10,11]. From this point of view, it is natural to apply in evolution theory different ideas of learning theory and vice versa. In particular, regularization, an important idea in learning, looks promising for evolution theory, as was shown at the end of the previous Section, universal regularization (by mutation rates) in learning problems of evolution gives universal distributions in genomics. The analogy between evolution and learning goes even further—in a discussion by R. Fisher [18], selection was considered as a random phenomenon (random weather conditions, etc.). These arguments look similar to the statistical learning theory (where training data are randomly chosen, see Appendix A.3). In a more standard discussion of evolution theory, training data are considered as fixed (selection is fixed).

The analogy between optimization and selection can be considered as an application in the learning of Darwinism, or the first evolutionary synthesis. Modern evolution theory is the population genetics, or the second evolutionary synthesis. In population genetics, an ensemble (population) instead of a single object is considered. One of the central achievements of population genetics is the explanation of purifying selection. Purifying selection was discussed by R. Fisher [18]; it is related to competition of different genotypes and prevents degradation of the fitness by mutations. Learning in population genetics can be defined as the convergence of the population of hypotheses to a peak around the minimum of the risk functional. The transition from learning a single hypothesis to learning a population of hypotheses is analogous to the transition from mechanics to statistical mechanics (where ensembles are studied). Using this analogy, we propose to discuss error catastrophe, or transition to ineffective purifying selection, as a model of overfitting in learning.

We formulate the learning model as the analog of the considered above generalization of Eigen’s model. Let $x_{f}\left(t\right)\geq 0$, $\sum_{f}x_{f}\left(t\right)=1$ be a normed distribution on the hypothesis space F of the learning system (the “space of genotypes” or hypotheses). Let us consider the analog of mutations in genetics—a list of partially defined maps $E=[e_{1},\cdots ,e_{n}]$, $e_{s}:F\to F$ of the hypothesis space. Hypotheses are generated from the initial hypothesis (in biology, ancestral genome) by an iterated application of hypothesis transformation operations (in biology, mutations).

The model of learning by population genetics is given by the following matrix Riccati equation (an analog of (1))

(11) $$\frac{d}{dt}x_{f}\left(t\right)=\sum_{g}Q_{fg}x_{g}\left(t\right)-x_{f}\left(t\right)\sum_{f,g}Q_{fg}x_{g}\left(t\right)$$

where mutation rates $Q_{fg}$, f≠g have the form (6) used in the discussed above generalization of Eigen’s model with the defined above mutations (hypotheses transformations) $E=[e_{1},\cdots ,e_{n}]$ and corresponding weights $w(e_{s})>0$ (efforts to perform mutations), diagonal matrix elements are given by (7), where *R* is the risk functional of the learning problem under consideration.

The discrete time analog of (11) is

$$x_{f}\left(t+1\right)=\frac{\sum_{g}Q_{fg}x_{g}\left(t\right)}{\sum_{f,g}Q_{fg}x_{g}\left(t\right)}.$$

One of the central problems in learning theory is overfitting, which is a strong dependence of the results of learning on the training sample—if the learning system is too complex it can overreact to small details of the data, hence a large subset of the hypothesis space contribute to learning. Therefore, overfitting is related to high entropy of the hypothesis space, to control overfitting a regularization is applied, see, in particular, VC theory [17].

In the above model of learning by population genetics, overfitting can be considered an error catastrophe, or transition to the regime of ineffective purifying selection, or divergence of the statistical sum (8), which gives the estimate for the Perron–Frobenius eigenvalue of the model as a matrix Riccati equation (actually, the estimate of the PF eigenvalue minus the diagonal matrix element in (A1)). Divergence of (8) means that the large subset of the space of genomes contribute to the population, and selection can not isolate the most fit genotype. This implies the divergence of (8) due to the large entropy of the hypothesis space F (the Boltzmann factor in (6) decays slowly with additional mutations). Convergence of (8) is provided if this decay is fast; this can be considered a regularization in the learning problem.

The condition of convergence of the statistical sum (8) can be satisfied for a wide choice of learning models and sufficiently low temperatures (large α). This condition is much less restrictive than the condition of the finite VC dimension in VC theory. This gives a criterion of the presence of overfitting in a population genetics-type learning problem. This criterion is a thermodynamic type effect and can be understood only if an ensemble (population) of learning systems is considered. The author does not claim that this statement about control of overfitting in learning by population genetics is mathematically proven, the idea is to exploit physical (and biological) intuition in the learning theory.

In the above discussion, we considered the fixed risk functional *R* (i.e., fixed training sample). In principle, one can vary the sample (use test data instead of training data); this will modify the risk functional *R* (selection) and diagonal matrix elements (7), but will not change the off-diagonal matrix elements (6) (mutations) and the statistical sum (8); hence, predictions on overfitting will be the same.

**Relation to “complexity as energy”**. The theory of “complexity as energy” was discussed by Yu.I. Manin [19]. In this approach, the Gibbs distribution with the Hamiltonian equal to the Kolmogorov complexity was applied to explain the power Zipf’s law of word frequency distribution in texts. The sum of weights of hypothesis generation operations in (6) can be discussed as a weighted upper bound for the Kolmogorov complexity of generation of a hypothesis. Therefore, statistical sum (8) is an example (of approximation) of the “complexity as energy” approach.

**Relation to GAN**. Generative Adversarial Network (or GAN) is a learning model which works by contest of two neural networks, generator and discriminator [20]. Modification and competition of networks at each step of the contest can be considered as analogs of mutation and selection correspondingly. From this point of view, this looks like a kind of the predator–prey model with mutations; moreover, GANs are described by minimax models similar to evolutionary game theory [21]. It is a general opinion of biologists that the predator–prey competition accelerates evolution greatly and this can be considered as an explanation why GANs are very successful. It looks like different models of population genetics might contribute to learning theory, in particular, the generalization of Eigen’s model introduced in the present paper. One can also mention genetic algorithms as an example of applications of biological ideas in learning.

**Summary**. In the present paper, we introduced a generalization of the Eigen’s model in population genetics and described the error catastrophe (transition to ineffective purifying selection) as a divergence of the Perron–Frobenius eigenvalue of the mutation–selection matrix of the model. The introduced model explains known patterns of genomic evolution. We propose to consider this population genetics model as a model of learning, where: the learning model is an ensemble (population) of learning models (a distribution on the hypothesis space); the risk functional in learning is described by fitness in population genetics; mutation rate matrix in population genetics corresponds to a set of hypothesis transformation operations and corresponding matrix of transformation (mutation) rates; learning reduces to convergence of the population of hypotheses to a peak around the minimum of the risk functional. Then, overfitting in learning can be described as the error catastrophe in population genetics, this criterion of overfitting can be understood only using ensembles (populations) of hypotheses.

## Data Availability

Not applicable.

## Appendix A

### Appendix A.1. Perron–Frobenius Theorem

Let $A=(a_{ij})$ be a square matrix with positive matrix elements, then:

(1) The largest in modulus eigenvalue *r* (Perron–Frobenius eigenvalue) is real and positive;
(2) This eigenvalue is simple (non-degenerate);
(3) There exists an eigenvector (Perron–Frobenius vector), corresponding to *r* with strictly positive coordinates, all other eigenvectors do not have this property;
(4) $\lim_{k\to \infty }\frac{A^{k}}{r^{k}}=P$, where *P* is a projection to the Perron–Frobenius vector;
(5) Eigenvalue *r* satisfies inequalities
  (A1) $$\min_{i}\sum_{j}a_{ij}\leq r\leq \max_{i}\sum_{j}a_{ij}.$$

For a matrix with non-negative matrix elements, the analogous properties are satisfied (these properties can be obtained as limits of the above properties, in particular the highest eigenvalue can be degenerate and some coordinates of the corresponding eigenvector can be zeros).

### Appendix A.2. Matrix Riccati Equation

In [22], an approach to analysis of texts based on matrix Riccati equations is discussed. Namely, the matrix is considered

$$A=\left(\begin{array}{cc} A_{1} & A_{2}\\ A_{3} & A_{4} \end{array}\right),$$

where $A_{4}$ is a (N−1)×(N−1)–matrix, $A_{1}$ is a number, $A_{2}$ and $A_{3}$ correspondingly are row and column of length N−1.

The corresponding map of the projective space $P^{N-1}$ is investigated

$$A:y_{m}=\left(\begin{array}{c} 1\\ x_{m} \end{array}\right)\mapsto y_{m+1}=\left(\begin{array}{c} 1\\ x_{m+1} \end{array}\right),\;x_{m+1}=\frac{A_{3}+A_{4}x_{m}}{A_{1}+A_{2}x_{m}}.$$

This discrete time dynamical system (iteration of the map above) can be considered as a discretization of the matrix Riccati equation

(A2) $$\frac{d}{dt}x\left(t\right)=A_{3}+A_{4}x\left(t\right)-x\left(t\right)A_{1}-x\left(t\right)A_{2}x\left(t\right).$$

The corresponding flow converges to a stationary point defined by the Perron–Frobenius theorem (under corresponding constraints for matrix *A*).

### Appendix A.3. Basic Definitions of the Statistical Learning Theory [17]

Learning theory discusses extracting patterns from data. In particular, the definition of a supervised learning problem is as follows: let us consider a training sample (i.e., a set of labeled data) $z_{i}=\left(x_{i},y_{i}\right)$, $x_{i}\in X$, $y_{i}\in Y$. We have to find a function (hypothesis) f:X→Y in the hypothesis space F related to the training sample.

Let p(x,y) be a probability distribution in X×Y. To evaluate a hypothesis, we will consider the loss (or risk) function V(f(x),y) with non-negative values. The expected risk functional is defined as an average of the risk function

$$R\left[f\right]=\int_{X\times Y}V\left(f\left(x\right),y\right)p\left(x,y\right)dxdy.$$

The problem of statistical learning is to find a hypothesis that gives the minimum of the risk functional

$$f=\arg\min_{h\in F}R\left[h\right].$$

The empirical risk functional

$$R_{\mathrm{emp}}\left[f,\mathrm{data}\right]=\frac{1}{n}\sum_{i=1}^{n}V\left(f\left(x_{i}\right),y_{i}\right),$$

where $\mathrm{data}=\left\{z_{i}\right\}=\left\{\left(x_{i},y_{i}\right)\right\}$, i=1,⋯,n is a training sample generated by the probability distribution, p(x,y), should approximate the expected risk functional.

The supervised learning problem is to find the optimal hypothesis defined by the training sample

$$f\left[\mathrm{data}\right]=\arg\min_{h\in F}R_{\mathrm{emp}}\left[h,\mathrm{data}\right].$$

**Classification problem**. Let $y_{i}=0,1$, a hypothesis *f* belongs to a family of characteristic functions (i.e., f(x)=0 or f(x)=1), and the risk function V(f(x),y)=|f(x)−y| is as follows: it is equal to zero for f(x)=y and to one otherwise. In this case, the empirical risk functional is given by the average number of errors of the risk function at the training sample:

$$R_{\mathrm{emp}}\left[f,\mathrm{data}\right]=\frac{1}{n}\sum_{i=1}^{n}\left|f\left(x_{i}\right)-y_{i}\right|.$$

**Problem of overfitting**. The following situation is possible: it is possible that the optimal hypothesis f[data] computed for the training sample data will give high values of the empirical risk functional if we would compute this functional for a control sample ${\mathrm{data}}^{\prime }$ different from data. This phenomenon is called overfitting. According to the Vapnik–Chervonenkis theory (or VC theory) [17], overfitting is explained by high entropy of the hypothesis space.

A general approach to control overfitting is the application of regularization: one can add a non-negative regularizing term depending on the hypothesis to the functional of empirical risk

(A3) $$H\left[f,\mathrm{data}\right]=R_{\mathrm{emp}}\left[f,\mathrm{data}\right]+Reg\left[f\right],$$

then, the learning problem will have the form

(A4) $$f_{Reg}\left[\mathrm{data}\right]=\arg\min_{h\in F}H\left[h,\mathrm{data}\right].$$

If a regularization term corresponds to some potential well in the hypothesis space, the optimization problem will be restricted to this well. In this way, the entropy of the hypothesis space will be limited.
